# Comparison of the Effects of Monounsaturated Fatty Acids and Polyunsaturated Fatty Acids on Liver Lipid Disorders in Obese Mice

**DOI:** 10.3390/nu15143200

**Published:** 2023-07-19

**Authors:** Wen Liu, Min Zhu, Meng Gong, Wen Zheng, Xin Zeng, Qing Zheng, Xiaoyu Li, Fudong Fu, Yingyi Chen, Jingqiu Cheng, Zhiyong Rao, Yanrong Lu, Younan Chen

**Affiliations:** 1 Department of Clinical Nutrition and Key Laboratory of Transplant Engineering and Immunology, NHFPC, Regenerative Medicine Research Center, West China Hospital, Sichuan University, Chengdu 610041, China; m13880308632@163.com (W.L.);; 2 Institutes for Systems Genetics, Frontiers Science Center for Disease-Related Molecular Network, West China Hospital, Sichuan University, Chengdu 610000, China; 3 Metabolomics and Proteomics Technology Platform, West China Hospital, Sichuan University, Chengdu 610041, China; 4 Department of Clinical Nutrition, West China Hospital, Sichuan University, Chengdu 610041, China

**Keywords:** obesity, insulin resistance, hepatic steatosis, unsaturated fatty acids, targeted lipidomics

## Abstract

Obesity is a recognized epidemic worldwide, and the accumulation of excess free saturated fatty acids (SFAs) in cells induces cellular lipotoxic damage and increases the risk of a wide spectrum of metabolic diseases including type 2 diabetes (T2D) and nonalcoholic fatty liver disease (NAFLD). Monounsaturated fatty acids (MUFAs) and polyunsaturated fatty acids (PUFAs) have been reported to combat SFA-induced cellular damage. However, the comparative studies of the two types of unsaturated fatty acids (UFAs) are still limited. We investigated the effects of different MUFAs and PUFAs in the human hepatocyte line L-02 cells in vitro, and in high-fat-diet (HFD)-induced obese C57BL/6 mice in vivo. The results of the in vitro study showed that SFAs induced significant cellular lipotoxic damage, but the combination of MUFAs/PUFAs with SFAs significantly improved the impaired cell viability. Particularly, oleic acid (OA) was superior to eicosapentaenoic acid (EPA), Docosahexaenoic acid (DHA), and arachidonic acid (AA) in terms of its anti-apoptotic effect and inhibition of endoplasmic reticulum (ER) stress. In vivo, both olive-oil-enriched (HFD + OO) and fish-oil-enriched high-fat diets (HFD + FO) reduced hepatic steatosis and improved insulin sensitivity in obese mice. However, FO induced an abnormal increase in serum aspartate aminotransferase (AST) and an increase in the oxidative stress indicator Malondialdehyde (MDA). Liver-targeted lipidomic analysis showed that liver lipid metabolites under the two types of UFA dietary interventions differed from the HFD group, modulating the abundance of some lipid metabolites such as triglycerides (TGs) and glycerophospholipids. Furthermore, the FO diet significantly increased the abundance of the associated FA 20:5 long-chain lipid metabolites, whereas the OO diet regulated the unsaturation of all fatty acids in general and increased the abundance of FA 18:1 in the overall lipid metabolites, especially TGs, which may primarily contribute to the FO, and OO drove protection in NAFLD.

## 1. Introduction

Obesity is a major health challenge for people worldwide as it greatly increases the risk of metabolic diseases such as type 2 diabetes (T2D), nonalcoholic fatty liver disease (NAFLD), hypertension, and hyperlipidemia [1]. Excessive food intake, especially high-calorie diets, is considered the greatest risk factor for obesity. With increasing energy intake, fat accumulates excessively in adipose tissue in the form of triglycerides (TGs), resulting in hypertrophy and the hyperplasia of adipocytes. The enhanced lipolysis of adipocytes releases more free saturated fatty acids (SFAs) into the peripheral blood circulation and promotes their deposition in non-adipose tissues, including the liver, heart, and pancreas, which is called “ectopic lipid deposition”. Under physiological conditions, small amounts of lipids in non-adipose tissues play an energetic role, mainly through the β-oxidation pathway, and when excess lipids accumulate in tissues, they disturb cellular lipid, glucose, and amino acid metabolism, thereby inducing cell dysfunction and programmed cell death [2]. The most abundant SFA in a high-fat diet (HFD) is palmitic acid (PA, 16:0), followed by stearic acid (SA, 18:0) [3]. It is well established that SFAs are the major cause of lipotoxic damage to tissues and cells, which induces a wide spectrum of homeostasis disturbances, including endoplasmic reticulum (ER) stress, oxidative stress, autophagy, and chronic inflammation by increasing the accumulation of lipid droplets and toxic metabolites such as ceramide. In the liver, the excess accumulation of SFAs disrupts hepatic glucolipid metabolism, contributing to liver insulin resistance (IR) and steatosis, and leading to the occurrence of NAFLD [4].

Monounsaturated fatty acids (MUFAs) and polyunsaturated fatty acids (PUFAs) are not only less cytotoxic than SFAs, but also potent in improving SFA-induced cellular damage, so they are considered useful for the prevention and supportive therapy of NAFLD and T2D [5,6,7]. Oleic acid (OA, 18:1, N = 9), with the highest physiological concentration of MUFAs in the human body, has a double bond at the ninth carbon atom at the methyl end of the fatty acid chain. A great number of studies have reported that OA has anti-inflammatory and immunomodulatory effects, and is able to improve peripheral IR, regulate liver enzymes, reduce hydrogen peroxide production, and alter membrane fluidity and membrane lipid peroxidation [8]. Obese patients on long-term MUFA diets, such as the Mediterranean diet and the olive-oil-rich diet, showed improvements in insulin sensitivity and a reduced risk of cardiovascular disease. Docosahexaenoic acid (DHA, 22:6, N = 3) and eicosapentaenoic acid (EPA, 20:5, N = 3) are N-3 long-chain PUFAs with the first double bond located on the third carbon atom at the methyl end of the fatty acid chain. N-3 PUFAs are widely recognized as a complementary nutrient for health, and studies have shown that N-3 PUFAs reduce the risk of a variety of diseases, including cardiovascular disease, NAFLD, hyperlipidemia, and even cancer [9,10]. A systematic review of the effects of SFAs, PUFAs, MUFAs, and carbohydrates on glucose–insulin homeostasis concluded that replacing 5% of the energy of SFAs with PUFAs significantly reduced blood glucose, HbA1c, C-peptide, and the IR index HOMA. Furthermore, by replacing either carbohydrates or SFAs, either MUFAs or PUFAs significantly improved the insulin secretion capacity [11].

However, in the LDL receptor KO mouse study, the addition of fish oil to an atherogenic HFD significantly reduced NAFLD scores associated with steatosis and inflammation, but no improvement was observed with olive oil (60 mL/kg) supplementation [12]. Although many studies have reported the benefits of UFAs in preventing and improving diabetes and regulating hepatic steatosis, there are still differences in the improvement effects of PUFAs or MUFAs in clinical trials, and even similar experiments have yielded different results. In addition, the mechanisms by which PUFAs or MUFAs interfere with lipotoxic injury and regulate lipid metabolism have not been fully elucidated, and the comparative studies of the two types of UFAs are still limited. Therefore, in this study, we aimed to comprehensively compare the effects and mechanisms of MUFAs and PUFAs, focusing on OA, EPA, and DHA, through a PA-challenged cell model in vitro and a HFD-induced NAFLD mouse model in vivo.

## 2. Materials and Methods

### 2.1. Fatty Acid Solutions

Eicosapentaenoic acid (EPA), Stearic acid (SA) (Sigma, Taufkirchen, Germany), Palmitic acid (PA), Oleic acid (OA), Linoleic acid (LA), Docosahexaenoic acid (DHA), Palmitoleic acid (POA), and Arachidonic acid (AA) (Aladdin, Shanghai, China) were dissolved in water-free ethanol (concentration: 100 mM) and combined with 20% (*w*/*v*) fatty-acid-free Bovine serum albumin (BSA) (Solarbio, Beijing, China), then incubated for 1 h at 50 °C to obtain a stock solution of 10 mM. BSA solvent control: Dilute 20% BSA to 18% BSA with anhydrous ethanol (BSA: 2.7 mM). The concentration of BSA in the fatty acid working solution/stock solution was the same as the BSA solvent control concentration. The concentration of BSA in the 100 μM fatty acid working solution was 27 μM.

### 2.2. Cell Culture

The human liver cell line L-02 was cultured in RPMI 1640 supplemented with 1% antibiotics (100 U/mL penicillin and 100 μg/mL streptomycin) and 10% fetal bovine serum (FBS). They were incubated at 37 °C and with 5% CO_2_ in a Lab-Line CO_2_ Incubator (Thermo, Waltham, MA, USA). The cells were treated with working fatty acid solutions prepared with fresh medium (2% FBS) for 24 h.

### 2.3. Cell Viability Detection

L-02 was cultured at 5000 cells/well in 96-well plates. After treatment, cell viability was measured using Cell Counting Kit-8 (CCK8, DOJINDO, Kumamoto, Japan).

### 2.4. Cell Apoptosis Detection

Cells were cultured overnight in a 6-well plate at 4 × 10^5^ cells/well. After treatment, cells are collected, and the apoptosis kit procedure was performed (BD, Franklin Lakes, NJ, USA). Apoptotic cells were detected via flow cytometry (FCM) and the numbers of early apoptotic cells and late apoptotic cells were counted.

### 2.5. ROS Detection

Cells were harvested and centrifuged at 400× *g* for 5 min. The cells were resuspended to 1 × 10^6^ cells/mL with 5 μM DCFH-DA working solution (Biyuntian, China) at 37 °C for 20 min, washed twice with PBS, and resuspended in 400 μL of PBS for machine analysis by FCM.

### 2.6. Western Blot Analysis

Cell protein extracts were separated by 10% SDS-PAGE gel and transferred to 0.2 μM PVDF membranes. After being blocked for 2 h in 5% skimmed milk, the membranes were subsequently probed by primary antibodies, including cleaved caspase-3 (CST, 9664S, MA, USA), β-actin (ABclonal, A17910, China), XBP-1 (Wanlei, WL00708, China), BIP (Absin, ab21685, China), BAX (Abcam, ab216494, UK), BCL-2 (Abcam, ab196495, UK), CHOP (Wanlei, WL00880, China), ATF4 (Huaan, ET1612-37, China), and ATF6 (Huaan, DF6009, China). Western Blot imaging was performed using the ChemiDoc™ Imaging System (Bio-Rad, CA, USA). The WB results were analyzed using ImageJ 1.53K software by dividing the grey value of the target protein by the grey value of the internal reference actin and then normalizing the data.

### 2.7. Real-Time PCR

RNA was isolated using Trizol reagent (Thermo, MA, USA), and reverse-transcribed into cDNA using a high-capacity cDNA Synthesis Kit (Vazyme, China). Real-time PCR was performed in a Bio-Rad qPCR Machine (CFX96, Bio-Rad, CA, USA) using the SYBR Green master mix (Vazyme, China). The data for both cellular and animal gene expression were calculated using β-actin as an internal reference and the 2^−ΔΔCt^ method was used to calculate the relative expression of genes. The sequences of primers are shown in Table 1.

### 2.8. Animals

Male C57BL/6 mice (6 weeks old; 18 ± 2 g) were housed in animal care facilities under controlled temperature (21~25 °C) and humidity (40~70%) with a 12 h light and dark cycle. Then, 56 mice were randomly divided into two groups, 14 in the normal control group and 42 in the high-fat diet (HFD) group. After 16 weeks, the HFD group was divided into three groups and fed HFD, HFD + FO (By-Health, China), and HFD + OO (Ouwei Li, China) feeds for an additional 12 weeks.

The 60% HFD, the customized fish oil supplement (HFD + FO), and the olive oil supplement (HFD + OO) diets (replacing 20% of the high-fat energy) were purchased from Jiangsu Xietong Company (Table 2). The body weights of these mice were monitored weekly, and their food intake was recorded accordingly. After treatment, the mice were sacrificed with an overnight fast, and blood and liver samples were collected. There were 14 different individuals in each group (n = 14). All the experimental animal procedures were approved by the Institutional Animal Care and Utilization Committee (IACUC) of Sichuan University. (Approval number: 20220119002).

### 2.9. Calculation of Liver Index

The liver index was calculated as follows:

Liver index = liver weight/body weight.

### 2.10. Biochemical Analysis

After fasting for 16 h, the whole blood of the mouse was centrifuged at 3000 rpm for 15 min at 4 °C to obtain serum samples. Alanine transaminase (ALT), aspartate aminotransferase (AST), triglyceride (TG), total cholesterol (TC), high-density lipoprotein cholesterol (HDL-C), lactate dehydrogenase (LDH), and low-density lipoprotein cholesterol (LDL-C) were detected using an auto-analyzer (Cobas 6000 C501, Roche, Basel, Switzerland).

### 2.11. Serum Insulin Detection

An ELISA kit (Mercodia Ultrasensitive Mouse Insulin ELISA, Sweden) was used to detect the fasting serum insulin levels in mice following the instructions of the manufacturer.

### 2.12. Fasting Blood Glucose (FBG), Glucose Tolerance Test (GTT), and Insulin Tolerance Test (ITT)

Animals were fasted overnight, and blood samples were obtained from the tail vein. Glucose concentrations were monitored using a pre-calibrated glucometer (Accu-chek Aviva, Roche, Basel, Switzerland).

To perform a GTT, mice were fasted overnight, and 2 g/kg body weight glucose was injected intraperitoneally. Blood glucose levels were measured at 0, 15, 30, 60, and 120 min after glucose injection via the collection of tail blood samples. The area under the curve (AUC) was assessed for each group from 0 to 120 min post glucose injection.

To perform an ITT, mice were fasted for 6 h, and 0.75 U/kg body weight of insulin was injected intraperitoneally. The subsequent steps were the same as the GTT experiment.

### 2.13. Electron Microscopy

Cells were collected and the supernatant was discarded, and a 3% glutaraldehyde fixed solution was slowly added. Liver tissue was stored in a 3% glutaraldehyde fixed solution. Sample processing and image collection were completed by Chengdu Lilai Medical Experimental Center.

### 2.14. Pathological Staining

Paraffin sections (3 μm) of liver tissue were dewaxed and rehydrated in different alcohol gradients for subsequent hematoxylin and eosin (HE) staining to visualize the histopathological changes via standard light microscopy. Liver sections (10 µm) frozen with 65% isopropanol were treated for 30 s, stained with Oil Red O (Nanjing Jian Cheng Bioengineering Institute, Nanjing, China) for 10 min, washed with 65% isopropanol for 12 s, and stained with hematoxylin. Oil red O staining was used to count the percentage of the red area using ImageJ 1.53K software.

### 2.15. Oxidoreductases Detection

Malondialdehyde (MDA), superoxide dismutase (SOD), glutathione (GSH), and lactate dehydrogenase (LDH) in liver tissues were detected to assess the antioxidant capacity of the liver. These were measured using a commercial kit (Nanjing Jiancheng Institute of Biological Engineering, Nanjing, China).

### 2.16. Triglyceride (TG) and Total Cholesterol (TC) Contents Detection

The contents of TG and TC in the livers were detected using commercial kits according to the kit’s instructions (Nanjing JianCheng Bioengineering Institute, Nanjing, China).

### 2.17. Targeted Lipidomics

After taking 10 mg (±5%) of liver tissues, 200 μL of dichloromethane: methanol = 1:1 (10 mM ammonium acetate) was added and the tissues were homogenized 3 times at 4 °C, then 150 μL of labeled methanol (methanol: Avanti IS = 145:5 (*v*/*v*)) was added. The extraction solution was added to 1000 μL methyl tert-butyl ether (MTBE), vortexed for 5 min at 4 °C, and kept away from light at room temperature for 30 min. Then, 250 μL of water (Mass spectrum grade) was added into the extract, swirled for 3 min, and then stood at room temperature for 10 min. Finally, the extract was centrifuged at 13,300 rpm for 10 min, and 800 μL of supernatant was taken and vacuum-dried for 2 h.

The sample tube was added with 200 μL of dichloromethane: MeOH = 1:1 (10 mM ammonium acetate), vortexed for 30 s, and centrifuged at 13,300 rpm for 10 min. Then, 80 μL of supernatant was loaded into 2 injection vials for loading onto the machine. Quality control (QC) samples: 30 μL of supernatant was taken from each sample, vortexed and mixed, and 80 μL was loaded into the injection vials. All samples were vortex-mixed for 15 min and then randomly loaded on an HPLC–MS/MS (AB Sciex Q-Trap 5500, MA, USA).

The raw mass spectrometry data were extracted using the commercial software MultiQuant 3.0.2 to extract peak areas, and then R software (version 3.6.1) was used to preprocess the extracted mass spectrometry data, including correcting the sample quality bias and removing low-quality ions (missing more than 50% in the QC sample, or missing more than 50% in the actual sample). Missing value filling (KNN algorithm), the removal of unstable ions (ions with relative deviation (RSD) >30% in all QC samples are filtered out), normalization (median), the log conversion of metabolic data, etc., were performed. Statistical analysis of the data included *p*-value calculation (Kruskal–Wallis with Dunn’s multiple comparisons test between multiple groups, Wilcoxon test between two groups. All lipid data were expressed as mean ± SD), principal component analysis (PCA), Partial Least Squares Discriminant Analysis (PLS-DA)/Orthogonal PLS-DA (OPLS-DA) analysis to screen for differential ions.

All metabolites were screened with VIP value ≥ 1 and FDR ≤ 0.05, and 467 metabolites were obtained. Then, the ChemSpider (https://www.chemspider.com/: accessed on 2 January 2023), HMDB (https://hmdb.ca/: accessed on 2 January 2023), and Metaboanalyst (https://www.metaboanalyst.ca/: accessed on 2 January 2020) were used for the 467 metabolites to convert the ID. Enrichment analysis was performed using MetaboAnalyst 5.0. Finally, differential metabolites were screened, FDR ≤ 0.01 and VIP value ≥ 1.3, and 24 differential phospholipid metabolites were screened.

### 2.18. Statistical Analysis

Histopathological data were analyzed using ImageJ 1.53K software. All cellular and animal data were expressed as mean ± SD. Statistical analyses were performed using GraphPad Prism 8.0. The Mann-Whitney test was used to compare the two groups and the Kruskal-Wallis with Dunn’s multiple comparisons test was used for the analysis of multiple groups. *p* < 0.05 was considered to be statistically significant.

## 3. Results

### 3.1. UFAs Improve SFA-Induced Cell Viability Damage and Apoptosis

**Figure 1:**
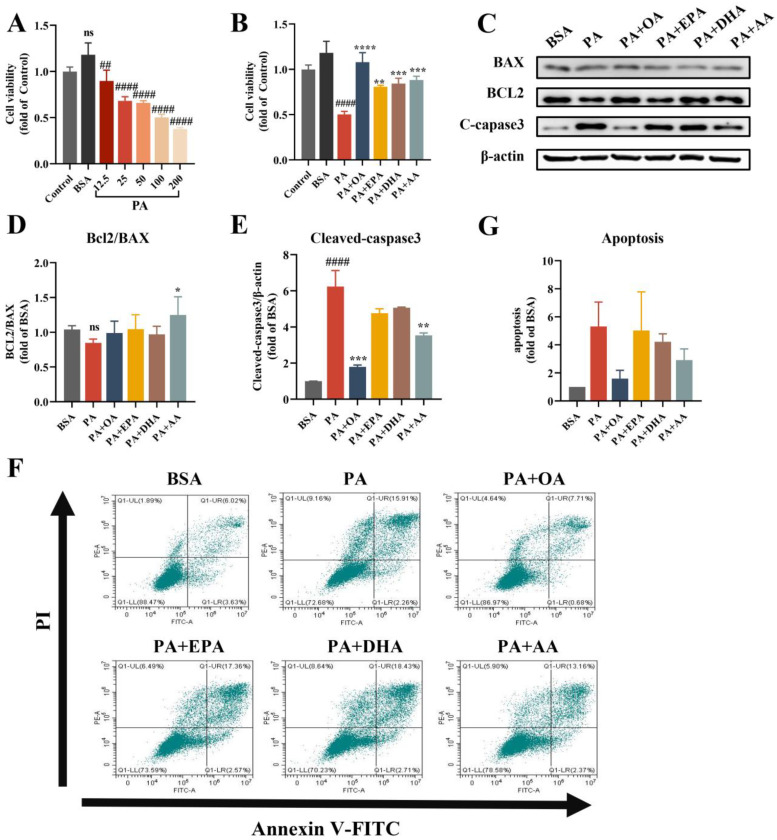
UFAs improve cell viability damage and apoptosis induced by SFAs in L-02 cells. (**A**) Effects of different PA concentrations (12.5 μM~200 μM) incubated for 24 h on the viability of L-02 cells. (BSA: 54 μM). (**B**) Effects of 100 μM OA and 25 μM EPA, DHA, and AA combined with 100 μM PA on the viability of L-02 cells. (BSA: 27 μM). (**C**) Protein expression levels of BCL2, BAX, and cleaved caspase-3. (BSA: 27 μM; PA: 100 μM; EPA, DHA and AA: 25 μM; OA: 100 μM). (**D**) Statistical analysis of BCL2/BAX protein expression. (**E**) Statistical analysis of cleaved caspase-3 protein expression. (**F**) Flow cytometry for apoptosis. (BSA: 27 μM; PA: 100 μM; EPA, DHA and AA: 25 M; OA: 100 μM). (**G**) Statistical results of apoptosis data. ns vs. Control/BSA group. ^##^ *p* < 0.01; ^####^ *p* < 0.0001 vs. BSA group. * *p* < 0.05, ** *p* < 0.01, *** *p* < 0.001, **** *p* < 0.0001 vs. PA group. Data are expressed as mean ± SD, (n ≥ 3). BSA as solvent control.

First, the effect of different concentrations of PA (16:0) treatment for 24 h on the viability of the human hepatocyte cell line L-02 was detected by CCK8. Cell viability decreased by approximately 10% after 12.5 μM PA treatment, by 31% and 34% after 25 μM and 50 μM PA treatment, respectively, and by 50% and 62% after 100 μM and 200 μM of PA treatment, respectively (Figure 1A). Because the fatty acid stock solution was prepared using fatty-acid-free Bovine serum albumin (BSA), BSA was chosen as the solvent control to eliminate the interference of BSA in the FFA experiments and to provide more accurate results. Similar results were obtained after the treatment of L-02 cells with the same concentrations of SA (18:0) (Figure S1A). We also compared the effects of the MUFAs OA (18:1) and POA (16:1), the N-3 PUFAs EPA (20:5) and DHA (22:6), and the N-6 PUFAs AA (20:4) and LA (18:2) on the viability of L-02 cells. No significant impairment of L-02 cell viability was observed after 24 h of treatment with different concentrations of UFAs (Figure S1B,C). The above results indicated that the cell viability of hepatocytes was damaged by SFAs in a concentration-dependent manner, while UFAs were much safer for cells.

According to the previous studies and physiological concentrations of various fatty acids [3,6,7,13,14,15,16], we chose 25 μM of PUFAs (EPA, DHA, and AA) and 100 μM of MUFAs (OA). To exclude the effect of concentration differences, we first used 25 μM or 100 μM of the same concentration of UFAs and 100 μM of PA to co-culture for 24 h. All UFAs effectively improved the cell viability in comparison with that of the PA group (increased from 50% to >80%, UFAs + PA groups vs. PA group, Figure S1D). The improvement in OA was best (Figure S1D and Figure 1B). Consistently, UFA-driven alleviation was also observed in SA-induced cell damage (Figure S1E). Therefore, considering the physiological concentrations range [3,14], 25 μM of PUFAs and 100 μM of OA were used for further experiments. These results strongly demonstrated that both MUFAs and N-3/N-6 PUFAs were able to effectively combat the SFA-induced impairment of hepatocytes.

We then examined the expression of the apoptosis-related proteins BCL2, BAX, and Cleaved-caspase3 via Western blotting (Figure 1C–E). Compared with the BSA group, PA significantly increased the expression of Cleaved caspase-3. After the combined treatment of PA and UFAs, the Cleaved caspase-3 decreased in each group, but only the PA + OA and PA + AA groups had significant differences compared to the PA group. Nevertheless, only the PA + AA group showed a significantly increased BCL2/BAX expression ratio compared to the PA group. Next, we detected apoptosis via flow cytometry with Annexin V FITC/PI staining. We counted the total percentage of early apoptotic (FITC^+^/PI^−^) and late apoptotic (FITC^+^/PI^+^) cells and performed a normalized analysis. Although the results of each group were not significantly different, OA displayed the best effect on reducing apoptotic cells (Figure 1F).

### 3.2. UFAs Attenuate PA-Induced ER Stress and Inflammatory Gene Expression

**Figure 2:**
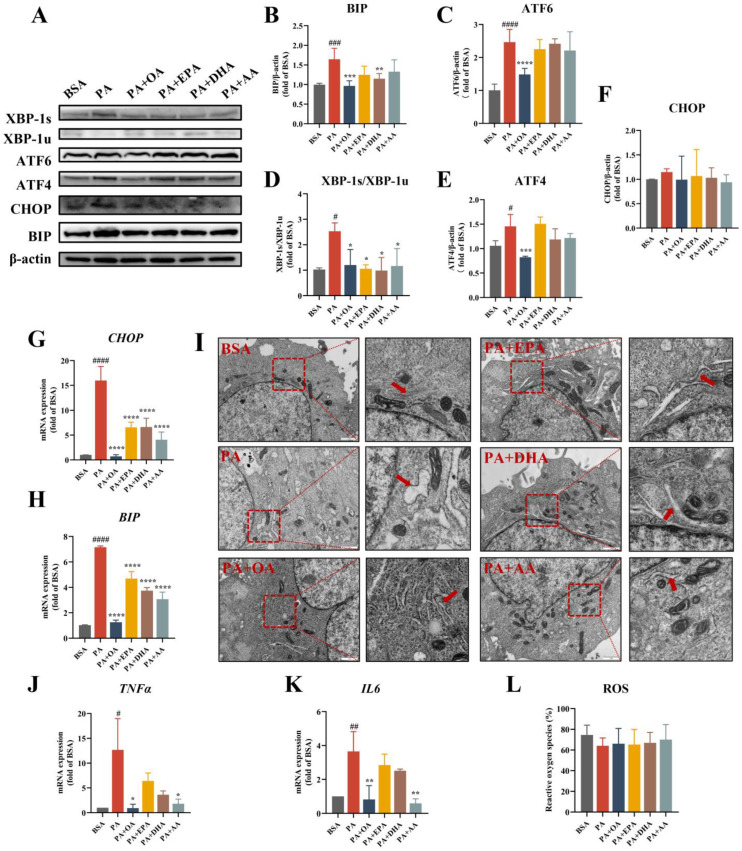
UFAs ameliorate PA-induced ER stress and the expression of inflammatory genes. (**A**) ER stress-related protein expression levels in L-02 cells. (BSA: 27 μM; PA: 100 μM; EPA, DHA and AA: 25 μM; OA: 100 μM). (**B**–**F**) Statistical analysis of protein expression levels in Figure (**A**). (**G**,**H**) Expression levels of the ER stress genes BIP and CHOP. (**I**) Effects of 100 μM OA and 25 μM EPA, DHA, AA combined with 100 μM PA on the morphology of the ER in L-02 cells. Red arrows indicate the ER. (BSA: 27 μM). (**J**,**K**) The expression levels of the inflammatory genes TNFα and IL-6 in L-02 cells after fatty acid treatment. (BSA: 27 μM; PA:100 μM; EPA, DHA and AA: 25 μM; OA: 100 μM). (**L**) The content of intracellular ROS detected by flow cytometry. (BSA: 27 μM; PA:100 μM; EPA, DHA and AA: 25 μM; OA: 100 μM). * *p* < 0.05, ** *p* < 0.01, *** *p* < 0.001, **** *p* < 0.0001 vs. PA group. ^#^ *p* < 0.05, ^##^ *p* < 0.01, ^###^ *p* < 0.001, ^####^ *p* < 0.0001 vs. BSA group. Data are expressed as the mean ± SD, (n ≥ 3). BSA as solvent control.

The effects of OA, EPA, DHA, and AA on the expression of ER stress-related proteins and genes were detected by Western blotting and real-time PCR. PA significantly upregulated the protein levels of BIP, ATF6, ATF4, and XBP-1s in L-02 cells (Figure 2A). The combination of each UFA and PA significantly decreased the ratio of XBP-1s/XBP-1u (Figure 2D), while only OA significantly reduced the protein levels of BIP, ATF6, and ATF4 (Figure 2B,C,E). In addition, the real-time PCR results suggested that all the UFA groups significantly improved the PA-induced high expressions of the ER stress genes *BIP* and *CHOP*, of which OA had the most outstanding effects (Figure 2G,H).

Next, we observed the changes in the subcellular structures in L-02 via transmission electron microscopy. After 100 μM of PA treatment for 24 h, the ER showed obvious expansion and swelling, and the normal lamellar folded structures disappeared. However, the four UFA groups significantly improved PA-induced ER swelling, and the PA + OA group had the best improvement, which could restore the normal ER morphology and structure (Figure 2I). These results suggested that OA may have a more important role in ameliorating ER stress in hepatocytes than PUFAs.

Furthermore, we detected the expression of the inflammatory genes *TNFα* and *IL6* via real-time PCR (Figure 2J,K). Compared with the BSA group, PA significantly increased the transcript levels of TNFα and IL-6, but only the PA + OA and PA + AA groups significantly downregulated the PA-induced high expression of inflammatory genes. Unexpectedly, EPA and DHA did not show a significant reduction in inflammatory gene expressions. Previous studies have reported that lipotoxicity induces oxidative stress. However, we detected no change in ROS production in L-02 cells under different fatty acid treatments using the fluorescent probe DCFH-DA (Figure 2L). We then examined the expression changes of genes related to lipid metabolism. We observed the significantly higher expression of stearoyl coenzyme A desaturase 1 (*SCD1*) after the PA challenge, but lower levels of *SCD1* in the other UFA combined groups (Figure S2D). Diacylglycerol O-acyltransferase 1 (*DGAT1*) had an expression trend as opposed to that of *SCD1* (Figure S2E). In addition, the fatty acid synthase (*FAS*), carnitine O-palmitoyltransferase 1 (*CPT1α*) genes, and acetyl coenzyme A carboxylase 1 (*ACC1*), which are associated with fatty acid synthesis and oxidation, did not produce significant differences in mRNA expression between the groups. To further explore the lipid metabolism affected by different fatty acids in hepatocytes, we then conducted targeted lipidomics studies on liver tissues.

### 3.3. Fish Oil/Olive Oil Supplementation Improves IR and Hepatic Steatosis in Obese Mice

**Figure 3:**
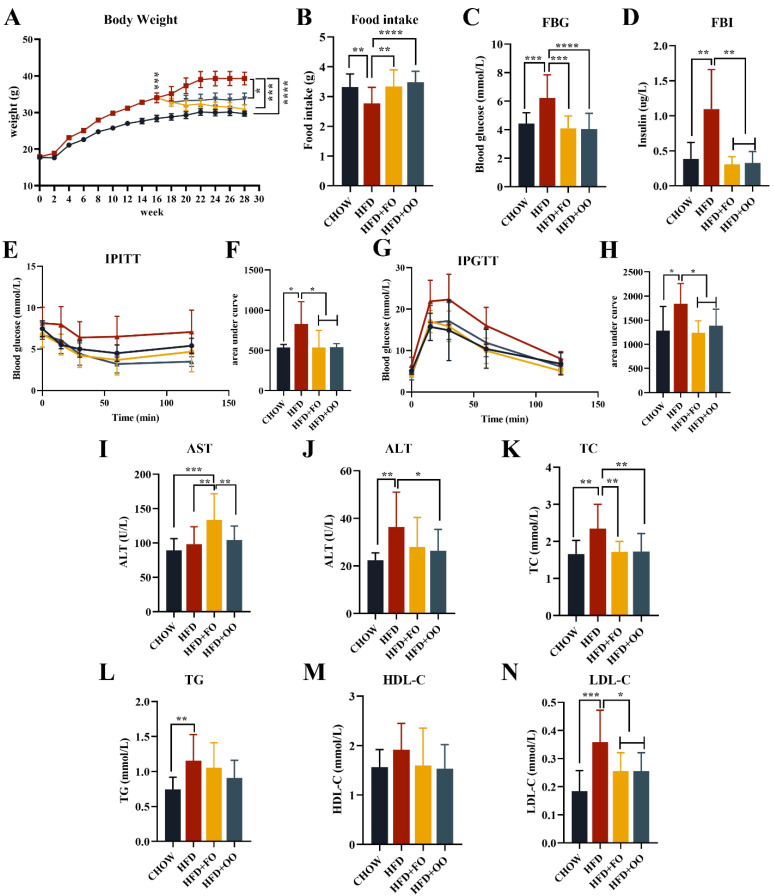
Results of body weight, insulin sensitivity, and blood biochemical indexes of mice fed with UFAs diets for 12 weeks. (**A**) Changes in body weight during the animal experimental study. (**B**) Differences in food intake (g/mouse/day) between groups. (**C**) FBG. (**D**) FBI. (**E**) IPGTT. (**F**) AUC of IPGTT. (**G**) IPITT. (**H**) AUC of IPITT. (**I**) AST. (**J**) ALT. (**K**) TC. (**L**) TG. (**M**) HDL-C. (**N**) LDL-C. * *p* < 0.05, ** *p* < 0.01, *** *p* < 0.001, **** *p* < 0.0001. Data are expressed as the mean ± SD, (n ≥ 13). Black line: CHOW group; Red line: HFD group; Yellow line: HFD+FO group; Blue group: HFD+OO group.

**Figure 4:**
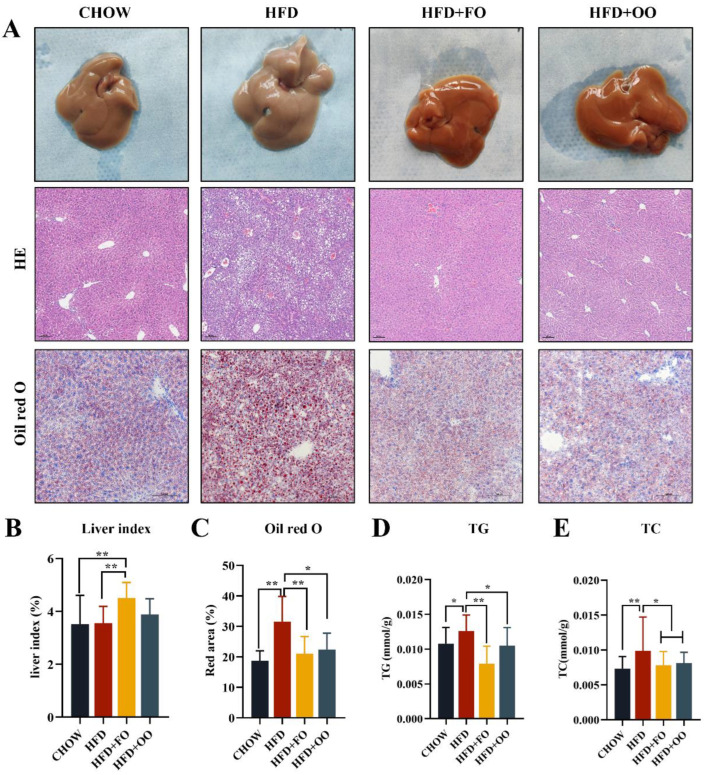
Histopathological results of the liver in mice fed with UFAs diets for 12 weeks. (**A**) Picture of mouse liver tissue, HE staining (100×) and Oil Red O staining (200×). (**B**) Liver index. (**C**) Statistics on the area of Oil Red O in (**A**). (**D**) The TG content of liver. (**E**) The TC content of liver. * *p* < 0.05, ** *p* < 0.01. Data are expressed as the mean ± SD, (n ≥ 5).

Male C57BL/6 mice were divided into two groups: CHOW and HFD. After being fed a 60% high-fat diet (HFD) for 16 weeks, the body weight and fasting blood glucose (FBG) of the mice in the HFD group were significantly increased compared to those of the mice in the CHOW group (Figure S3A,B), and impaired glucose tolerance (GTT) and insulin tolerance (ITT) were also found (Figure S3C–F). Then, the HFD group was divided into three groups and fed with HFD, HFD + FO, and HFD + OO for an additional 12 weeks. At the ending time, the body weights of the HFD + FO and HFD + OO groups were significantly lower than that of the HFD group (Figure 3A). This difference in body weight was not related to energy intake, as the HFD group ate significantly less than the other three groups (Figure 3B). In addition, both FBG and fasting blood insulin (FBI) levels were significantly decreased in the HFD + FO and HFD + OO groups compared to the HFD group (Figure 3C,D). More importantly, the UFA diets improved the obesity-induced impairment of intraperitoneal GTT and ITT (Figure 3E–H), indicating their beneficial role in insulin sensitivity. The biochemical parameter results also showed that both the HFD + OO and HFD + FO groups had significantly reduced serum TC and LDL-C levels compared to the HFD group (Figure 3K,N). In terms of the liver function indexes, only the HFD + OO group displayed significantly reduced serum ALT levels (Figure 3J). It is worth noting that the HFD + FO group slightly increased the serum AST levels (Figure 3I) and the liver index in comparison with the HFD group (Figure 4B).

The morphology of the livers showed a pale color in the HFD group, while the color of the livers returned to normal after the UFA diet intervention. HE staining and Oil Red O staining was performed on liver tissues to assess the pathological abnormality of the liver. Both the HFD + FO and HFD + OO groups showed a significant alleviation in liver steatosis compared to the HFD group (Figure 4A,C), and both lipid deposition and macrovesicular steatosis were remarkably alleviated. The results of the liver tissue TG and TC assay were consistent with the histological results, showing significantly reduced liver TG and TC levels in the HFD + FO and HFD + OO groups (Figure 4D,E). Summarily, both the HFD + FO and HFD + OO groups exhibited improved HFD-induced hyperlipidemia, hepatic steatosis, and insulin resistance (IR) in obese mice. Among them, HFD + OO seems to have better effects than HFD + FO in improving liver function.

### 3.4. Fish Oil/Olive Oil Ameliorates HFD-Induced ER Stress in the Livers of Mice

**Figure 5:**
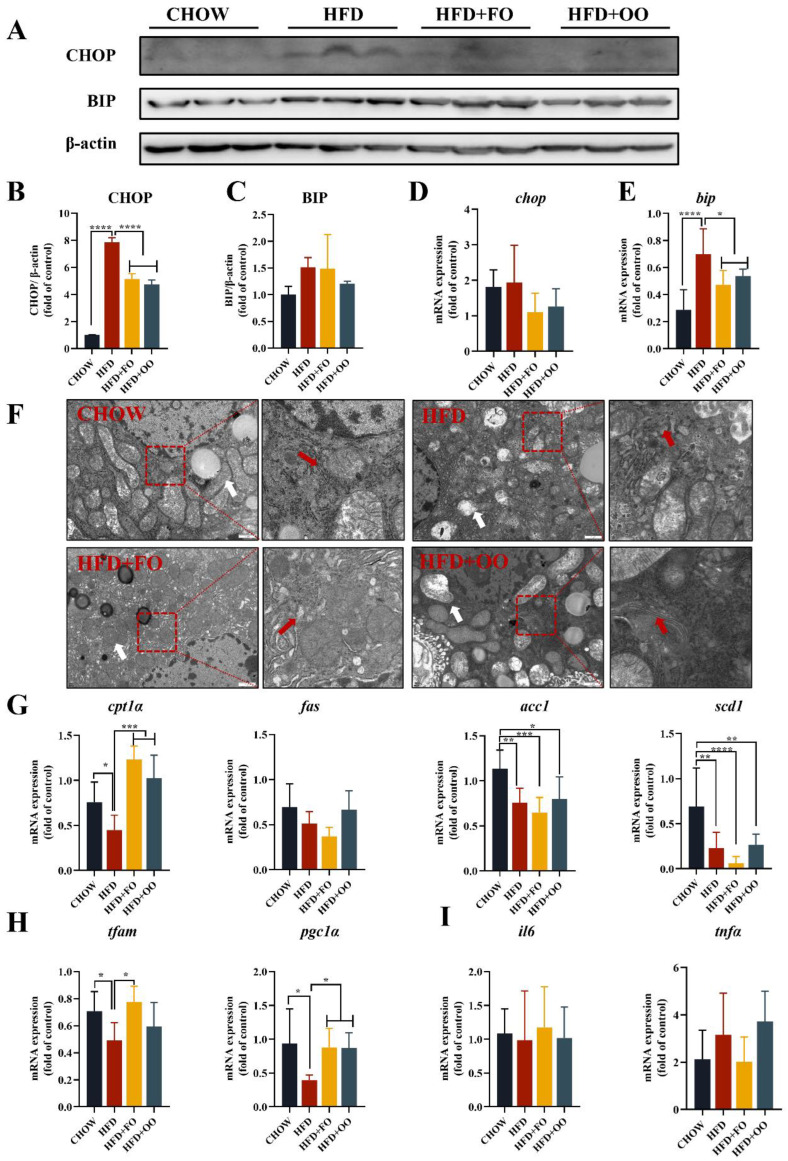
Protein and mRNA results of the liver in mice fed with UFA diets for 12 weeks. (**A**) The expression level of ER stress-related protein in the liver of mice. (**B**,**C**) Statistical analysis of protein expression levels in Figure (**A**). (**D**,**E**) Expression levels of the ER stress genes BIP and CHOP in the livers of mice. (**F**) The morphology of mouse liver tissue (red arrows indicate the ER and white arrows indicate mitochondria). (**G**) Lipid metabolism genes: CPT1α, FAS, ACC1, and SCD1. (**H**) Mitochondrial biogenesis-related genes PGC-1α and TFAM. (**I**) Inflammatory genes: TNFα and IL6. * *p* < 0.05, ** *p* < 0.01, *** *p* < 0.001, **** *p* < 0.0001. Data are expressed as the mean ± SD, (n ≥ 5).

We examined the protein and gene expression levels of BIP and CHOP via Western blotting and real-time PCR (Figure 5A–E). The protein levels of BIP were not significantly different between groups (Figure 5C), but at the mRNA level, *Bip* expression was significantly reduced in the HFD + FO and HFD + OO groups (Figure 5E). CHOP expression was significantly different at the protein level, with a significant elevation in the HFD group, while both HFD + FO and HFD + OO interventions significantly reduced CHOP expression (Figure 5B), but not at the mRNA level between groups (Figure 5D). We further observed the subcellular structures of the liver tissues in the four groups via transmission electron microscopy, in which the ER in the HFD group showed severe dilatation, swelling and the loss of normal lamellar folding structures, and the mitochondria showed a severely abnormal structure of rounded swelling and a loss of cristae. Both UFA diets improved ER morphology, with HFD + FO showing the best improvement in mitochondrial damage (Figure 5F).

We then examined the expression of genes related to lipid metabolism, mitochondrial function, and inflammation in mouse liver tissue. The expression levels of *cpt1α* for lipid oxidation and *pgc1a* for mitochondrial function were significantly increased in the HFD + FO and HFD + OO groups compared to the HFD group (Figure 5G,H), while inflammatory genes were not significantly different between groups (Figure 5I).

### 3.5. UFA Diets Regulated the Disturbed Lipid Metabolism Induced in Obese Mice

We used lipidomics to explore the effects of different fatty acid diets on lipid metabolites in the liver. In the principal component analysis (PCA) of Quality control (QC) in the positive and negative ion modes, the distribution of QC samples clustered together, indicating the stability and reproducibility of the lipidomic analysis (Figure S4A,B). The partial least squares discriminant analysis (PLS-DA) results showed that the CHOW group, HFD group, HFD + FO group, and HFD + OO group had significant separation degrees, indicating that there were distinct differences in the overall lipid metabolism among all groups and good reproducibility within groups (Figure S4C).

**Figure 6:**
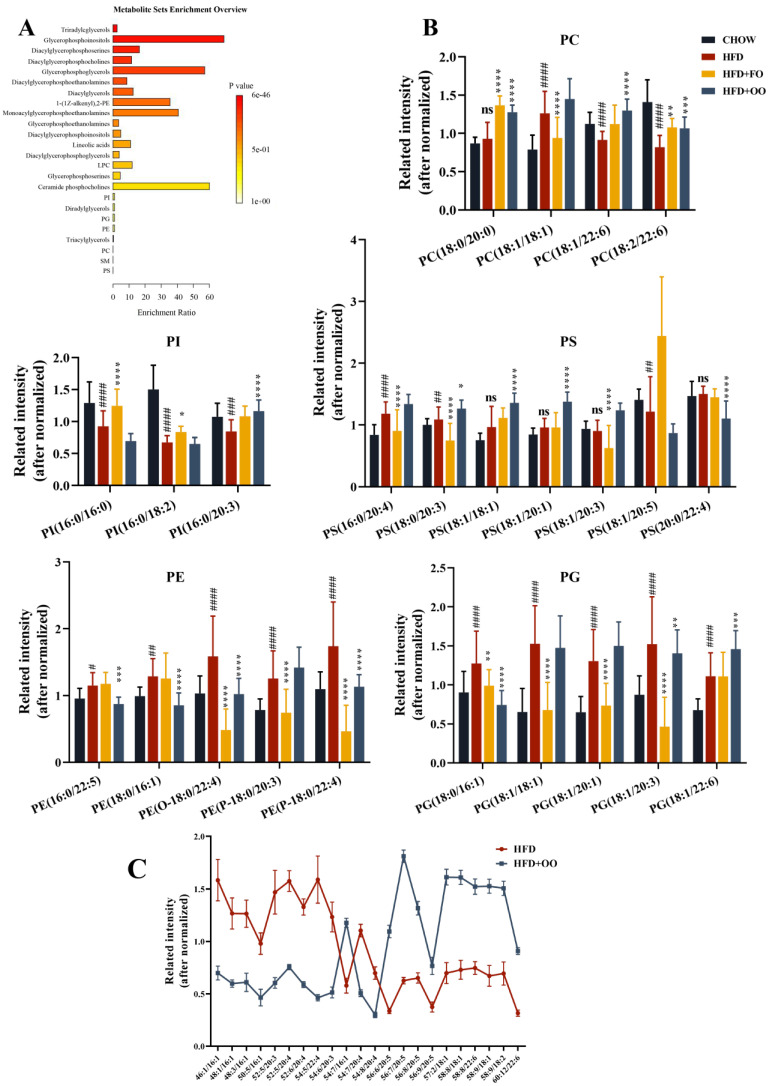
UFA diets regulated the disturbed metabolism induced by HFD in mice. (**A**) Enrichment analysis of significantly altered lipid metabolites. (**B**) Significant lipid metabolites. (**C**) TG-significant lipids between the HFD group and the HFD + OO group. ns vs. CHOW group. * *p* < 0.05, ** *p* < 0.01, *** *p* < 0.001, **** *p* < 0.0001 vs. HFD group. ^#^ *p* < 0.05, ^##^ *p* < 0.01, ^###^ *p* < 0.001, ^####^ *p* < 0.0001 vs. CHOW group. Data are expressed as the mean ± SD, (n ≥ 13). PI, Phosphatidylinositol; PS, Phosphatidylserine; PE, Phosphatidylethanolamine; PC, Phosphatidylcholine; PG, Phosphatidylglycerol.

An enrichment analysis of the lipid metabolites with significant differences was performed using the enrichment analysis module in MetaboAnalyst 5.0 (Figure 6A). In addition to glycerol esters, various types of lipids, including glycerophospholipids, lysophospholipids, etc., were enriched in the differential analysis (Figure 6A). Liver is rich in TGs, but the elucidation of the biological function of individual lipids is challenging due to different combinations of the acyl chain length, the number of double bonds, cis–trans, and acyl chain binding positions. In addition to TGs, the glycerophospholipid metabolism may play an important role in the development of NAFLD and in the protective effect of fish oil and olive oil. Therefore, we analyzed a great number of glycerophospholipid metabolites with significant differences (VIP ≥ 1.3). The 24 selected metabolites were categorized as phosphatidylinositol (PI) (3), phosphatidylserine (PS) (7), phosphatidylethanolamine (PE) (5), phosphatidylcholine (PC) (4) and phosphatidylglycerol (PG) (5). Compared to the CHOW group, the HFD group had upregulated PC (18:1/18:1), PE (16:0/22:5), PE (18:0/16:1), PE (O-18:0/22:4), PE (P-18:0/20:3), PE (P-18:0/22:4), PG (18:0/16:1), PG (18:1/18:1), PG (18:1/20:1), PG (18:1/20:3), PG (18:1/22:6), PS (16:0/20:4), PS (18:0/20:3), and PS (18:1/20:5) levels. Nevertheless, the HFD group had downregulated PC (18:1/22:6), PC (18:2/22:6), PI (16:0/16:0), PI (16:0/18:2), and PI (16:0/20:3) levels. Fish oil and olive oil showed consistent regulatory effects on the interventions for PC (18:2/22:6), PE (O-18:0/22:4), PE (P-18:0/22:4), PG (18:0/16:1), and PG (18:1/20:3) in the HFD + FO and HFD + OO groups (Figure 6B). However, for other pairs of lipid metabolites, FO and OO showed quite different modulatory effects. These results suggested that the glycerophospholipid metabolism may play an important role in the development of NAFLD, as well as the protective effects of fish oil and olive oil.

In addition, when we processed the TG data, we found that the HFD + OO group had distinctly more long-chain FAs and more double bonds in TGs than the HFD group after the olive oil diet intervention (Figure 6C), which suggested that the regulation of the chain length and the unsaturation of fatty acids may be critical mechanisms underlying olive-oil-mediated protection.

## 4. Discussion

### 4.1. UFAs Ameliorate SFA-Induced Lipotoxicity

In our experiments, human hepatocyte L-02 cells were treated with the saturated fatty acids PA and SA, and the cell viability was found to decrease with increasing concentrations. In contrast, the unsaturated fatty acids, including MUFAs, N-3 PUFAs, and N-6 PUFAs, were not found to significantly impair cell viability even at relatively high concentrations, and all of them displayed antagonistic effects toward SFA-induced lipotoxicity, which agrees with the previous results reported by other researchers. In our previous work, we also found that the hepatoma cell line HepG2, rat β-cell line INS-1E, and human endothelial cells HUVECs were impaired by PA in a dose-dependent manner, and that OA was able to restore cell viability [5,17,18]. To compare the ameliorative effects of MUFAs and PUFAs on lipotoxicity, we chose the N-3 PUFAs, EPA and DHA, and the N-6 PUFAs AA. Though we chose AA to represent N-6 PUFAs and verified its protective role in vitro, the literature reports the pro-inflammatory effect of AA and the potential causal relationship that AA may have with NAFLD [19,20], so we did not include AA in the animal experiments. Regarding the concentration of PUFAs used in the cell experiments, we referred to previous research, in which EPA, DHA, and AA were mostly used at a concentration of 20 μM in vitro [7,14]. Another cell experiment with DHA supplemented at 25 μM concentrations is reported to be close to the physiological plasma concentrations (4 g/d) of Wistar rats treated with fish oil for 60 days [15]. Therefore, the final concentration of PUFAs was 25 μM in most of our cell experiments. Regarding the concentration selection of MUFAs (OA), we refer to the previous laboratory studies and literature. At first, OA is the UFA with the highest physiological concentration in the human body, and the physiological concentration can reach 647 μM [3]. OA (300 μM) improved the apoptosis and ER stress induced by PA (300 μM) in HepG2 [13]. OA (200 μM) alleviates the ER stress and pyroptosis induced by PA (400 μM) in HepG2 [16]. NES2Y cells (human pancreatic beta cell line) were treated with different fatty acids, such as POA and OA, and did not affect the cell viability at concentrations up to 3 mM [6]. Considering the physiological concentration range, the same treatment concentration as PA was finally selected (OA: 100 μM).

It is well established that SFAs induce ER stress, mitochondrial dysfunction, and oxidative stress. In mammals, ER calcium depletion, resulting from alterations in the lipid composition of cell membranes, is thought to promote unfolded protein stress by interfering with the calcium-dependent chaperones and enzymes required for protein folding, thereby promoting unfolded protein stress through conventional transmission [21,22]. When unfolded or misfolded proteins accumulate in the ER, ER stress triggers the activation of unfolded protein response (UPR), and BIP is released, activating double-strand-dependent protein kinase ER kinase (PERK), inositol demand kinase 1α (IRE1α), and transcription factor 6 α (ATF6 α), and their downstream signaling pathways; therefore, the increased expression of BIP is also considered an important indicator of ER stress [23]. In the present study, we found that UFAs ameliorated SFA-induced ER stress in L-02 cells. PA increased the expression of the marker proteins of the three pathways to varying degrees. Additionally, an increase in the ratio of XBP-1s/XBP-1u and the swelling of the ER were also observed in L-02 cells, which is consistent with our previous experiments inINS-1E cells [17]. IRE1α-XBP1 acts as an adaptive signaling pathway and the UPR-mediated overexpression of XBP-1s can enhance the activity of the cytidine diphosphate choline (CDP-choline) pathway to synthesize phosphatidylcholine, the major phospholipid on the mammalian ER membrane that promotes membrane expansion and induces ER biosynthesis [24]. SFAs promote ER swelling by inducing ER stress and activating the IRE1α-XBP-1 pathway. When comparing the different roles of UFAs in regulating ER stress, our results clearly indicated that OA has the best suppressive effect in comparison with PUFAs. In an experiment in which pancreatic exocrine acinar cells were treated with different concentrations (200~500 mM) of different fatty acids (SFAs, MUFAs, and PUFAs) for 24 h, PA significantly increased the transcript levels of UPR (XBP1, CHOP, and BIP), and enhanced the time-dependent nuclear translocation of XBP1. PUFAs (DHA) cause an increase in the milder markers of ER stress, while MUFAs (OA) attenuate the ER stress response [25]. As shown in Figure S2, UFAs affected the expression of *SCD1* and *DGAT*, and it has been reported that there is a bias of endogenous MUFAs in TGs synthesis [26]. Perhaps OA is more beneficial in promoting the synthesis of inert TGs and improving SFA-induced ERs.

It has also been reported that 30 μM DHA at a physiological or nutritional level could improve IR and the expression of inflammatory genes (TNFa and IL6) in C2C12 cells induced by palmitate (500 μM) [27]. However, in our experiment, 25 μM EPA and DHA did not reduce the high expression of inflammatory genes in L-02 cells induced by 100 μM of PA. This may be related to a model of cellular lipotoxicity, which did not induce severe inflammation, and we did not detect levels of many other important inflammatory cytokines. Therefore, further validation regarding the anti-inflammatory effects of EPA and DHA is needed. However, unexpectedly, AA in L-02 cells significantly decreased the expression of inflammatory genes. It has been reported in the literature that AA at 25 μM improved apoptosis in W256 carcinosarcoma cells [28], and was effective in reducing the adipocyte inflammation induced by HFD in obese mice [29]. Therefore, the effects of AA need to be further investigated.

### 4.2. UFA Diet Improves IRand Hepatic Steatosis in Obese Mice

In a systematic review and meta-analysis, in healthy adults, replacing 5% of the energy of SFAs with PUFAs significantly reduced blood glucose, glycated hemoglobin, C-peptide levels, and the HOMA index. Whether replacing carbohydrates, SFAs, or MUFAs, PUFAs significantly improved insulin secretion [11]. In LDL receptor KO mice, the addition of fish oil or olive oil (60 mL/kg) to an atherogenic HFD significantly reduced the NAFLD scores associated with steatosis and inflammation, whereas olive oil had no improving effect [12]. In vivo human trials, as well as some in vitro animal studies, have provided experimental results demonstrating that fish oil appears to have superior effects on improving blood glucose, enhancing insulin sensitivity, and regulating blood lipids.

In our study, both the olive oil diet and the fish oil diet improved insulin resistance, significantly improved FBG and FBI, and enhanced insulin sensitivity in obese mice induced by an HFD. From the experimental results, neither the fish oil nor olive oil diets affected the mice’s food intake. The liver index of the mice in the HFD + FO group was significantly higher than the other three groups, which may be related to the fact that fish oil reduces body weight. In a previous clinical trial, supplementation with N-3 PUFAs significantly reduced the body mass index of patients with NAFLD [30]. However, high concentrations of fish oil may induce hepatic lipid peroxidation damage and elevate serum liver damage markers. We found that the hepatic Glutathione (GSH) level in the HFD group increased significantly, while the GSH level in the HFD + FO group and HFD + OO group decreased (Figure S5A). We speculate that the reason might be the compensatory increase in GSH in the HFD group. The level of malondialdehyde (MDA) indirectly reflects the severity of free radical attacks on cells. The hepatic MDA values in the HFD + FO group were significantly higher than those in the other three groups (Figure S5B). Fish oil is rich in long-chain polyunsaturated fatty acids (LPUFAs), which are highly sensitive to free radicals and other active oxidation groups, producing lipid peroxy radicals and oxidizing lipids. MDA is one of the end products of lipid peroxidation and is widely used to evaluate lipid peroxidation. Fish oil promotes the high expression of MDA in liver tissue, which may be related to the damage of lipid peroxidation, but this does not conflict with the need for PUFAs rich in UFAs in mitochondria mentioned later. A delicate balance needs to be struck to coordinate an improvement in the mitochondrial membrane fluidity due to the intake of PUFAs and the oxidative stress damage caused by LPUFAs [31]. The level of superoxide dismutase (SOD) activity indirectly reflects the body’s ability to scavenge oxygen-free radicals, and lactate dehydrogenase is an important enzyme in the process of glycolysis. Hepatocyte injury caused by any reason can lead to an increase in lactate dehydrogenase (LDH) activity. However, the levels of hepatic LDH and SOD did not differ among the four groups (Figure S5C,D).

As the high-dose fish oil diet induced lipid peroxidation damage, we also performed a comparative experiment with fish oil and olive oil replacing 5% of HFD energy, where the low dose of fish oil did not increase liver MDA levels (Figure S6N). However, in the low-dose substitution experiment, the two UFA diets did not improve FBG, FBI, and liver steatosis, only GTT, and in addition, the OA diet significantly reduced AST and ALT levels (Figure S6).

### 4.3. Targeted Lipidomics

In the targeted liposome analysis of the liver tissues of the four groups of mice, the results provide more evidence that fish oil and olive oil modulate NAFLD. In our analysis, we have paid more attention to glycerophospholipids, including PS, PI, PE, PG, and PC, since phospholipids are key components of the cellular lipid bilayer, are involved in a wide spectrum of cellular metabolisms and signaling pathways, and are more easily detected due to their high abundance.

Our results indicated that both types of UFAs differentially modulated HFD-induced lipid disorders after dietary intervention. The EPA intake from fish oil promoted much higher levels of FA20:5 in glycerophospholipids than in the other three groups (Figure S4D). The level of FA18:1 was also significantly higher in the olive oil group than in the other three groups (Figure 6B). This is related to the structure of the fatty acids carried in the corresponding diets, and suggests that both olive oil and fish oil modulate glycerophospholipids, but affect different lipid metabolites. Glycerophospholipids act as a highly synthetic subclass of lipids in the mitochondria-associated ER membrane (MAM), and the maintenance of the ER, mitochondria, and their MAM is essential for the regulation of cellular metabolism and the maintenance of energy homeostasis. In our experiment, both HFD + FO and HFD + OO diets improved the ER stress induced by HFD, but the maintenance of the mitochondrial organelle morphology by HFD + FO was more obvious. In the literature, the content of PUFAs in mitochondrial membranes is reported to be higher than that in their whole-tissue membranes, and the regulatory role of N-3 PUFAs may be related to the need for mitochondria to take up PUFAs to improve membrane fluidity [31].

Another experiment showed improved fatty acid beta oxidation after the ingestion of N-3 PUFAs, associated with DHA incorporation in the mitochondrial membrane [32]. In a double-blind, randomized, placebo-controlled trial, NASH patients received N-3 PUFAs for 6 months. Glycerophospholipid levels were significantly increased after N-3 PUFA treatment, and ER and mitochondrial function were improved. A high ratio of N-6/N-3 PUFAs can induce ER stress and is associated with disease pathology in NASH. Therefore, N-3 PUFAs regulate common pathways linking lipid metabolism, ER, and mitochondrial function [33], in relation to their ability to alter the lipid composition of subcellular organelle membranes [34].

Studies have observed that different types of FAs, especially PC, TG, and CE, become shorter, have fewer double bonds, and are rich in SFAs after intervention with an HFD. In contrast, after the Mediterranean diet intervention, MUFA-rich FAs become longer and have more double bonds [35]. This is consistent with our findings demonstrating that the unsaturation of long-chain fatty acids in TG increased after olive oil intervention (Figure 6C). In addition, in patients with hypercholesterolemia, compared with diets rich in SFAs or PUFAs, MUFA-rich diets change the length of phospholipids embedded in HDL, which in turn promotes changes in HDL monolayer fluidity and ultimately enhances HDL cholesterol outflow (ChE). Olive oil intake also changes the FA distribution of PC on the surface of HDL, increases MUFAs, and decreases SFAs. These changes are thought to be due to different fatty acid compositions in the dietary fat intake, especially olive oil with high levels of MUFAs (oleic acid). Among the different kinds of lipids, especially in high-density lipoprotein particles, the largest increase in FA is FA18:1 [36]. Therefore, in conjunction with experiments and the literature, we hypothesize that olive oil may regulate lipid metabolism in the liver by modulating the overall unsaturation of the fatty acid chain, thereby reducing the level of saturated free fatty acids, and enhancing the fluidity of lipoprotein membranes.

## 5. Conclusions

Our study demonstrates that SFAs induced significant cellular lipotoxic damage, but that UFAs had no significant cytotoxicity in the physiological concentration range in hepatocytes. The combination of MUFAs/PUFAs with SFAs significantly improved the impaired cell viability. The molecular mechanisms may be related to their ability to alleviate ER stress, reduce cell inflammation and decrease apoptosis. Importantly, we demonstrated that OA is superior to EPA, DHA, and AA in terms of its anti-apoptotic effects and inhibition of ER stress. In an HFD-induced metabolic disorder model, replacing 20% HFD with UFAs (fish oil or olive oil) improved the biochemical parameters, alleviated hepatic steatosis, and improved insulin sensitivity in obese mice. Fish oil improved hepatic steatosis, but was accompanied by an abnormal increase in serum AST levels and an increase in the oxidative stress indicator MDA, which may be related to its richness in long-chain fatty acids and increased risk of peroxidative lipid peroxidative damage. Targeted lipidomic analysis showed that both classes of UFAs regulated hepatic lipid metabolism. The main lipid metabolites involved were triglycerides, as well as glycerophospholipids. Particularly, the FO diet significantly increased the abundance of related FA 20:5 long-chain lipid metabolites, which may contribute to its ability to improve ER and mitochondrial function. On the other hand, olive oil, being rich in MUFAs, regulates the unsaturation of all fatty acids in general and increases the abundance of FA18:1 in general and TGs in particular. These data may provide new insights into dietary intervention in NAFLD and other metabolic disorders.

## Figures and Tables

**Table 1:** Primer sequences for qPCR.

Gene	Sequence
*H-CHOP-F*	CACTCTCCAGATTCCAGTCAG
*H-CHOP-R*	AGCCGTTCATTCTCTTCAGC
*H-BIP-F*	AAGAACCAGCTCCAATTGCA
*H-BIP-R*	CACCTTGAACGGCACGAACT
*H-IL6-F*	GAAAGCAGCAAAGAGGCA
*H-IL6-R*	CACCAAGTTGAGGGAATGA
*H-TNFα-F*	ACCTCCTCTCTGCCATCAAG
*H-TNFα-R*	GAGTCGATCACCCTTCTCCA
*H-SCD1-F*	CCTGGTTTCACTTGGAGCTGTG
*H-SCD1-R*	TGTGGTGAAGTTGATGTGCCAGC
*H-ACTIN-F*	CCACGAAACTACCTTCAACTCC
*H-ACTIN-R*	GTGATCTCCTTCTGCATCCTGT
*H-FAS-F*	GGACCCAGAATACCAAGTGCAG
*H-FAS-R*	GGACCCAGAATACCAAGTGCAG
*H-DGAT1-F*	CACAGAGGCCACAGAAGTGA
*H-DGAT1-R*	AGGGCAGATACCTCCAGACA
*H-CPT1α-F*	GATCCTGGACAATACCTCGGAG
*H- CPT1α-R*	CTCCACAGCATCAAGAGACTGC
*H-ACC1-F*	TTCACTCCACCTTGTCAGCGGA
*H-ACC1-R*	GTCAGAGAAGCAGCCCATCACT
*M-bip-F*	A TGA TGAAGTTCACTGTGGTGG
*M-bip-R*	CTGATCGTTGGCTATGATCTCC
*M-actin-F*	GTGGGAATGGGTCAGAAGGA
*M-actin-R*	CTTCTCCATGTCGTCCCAGT
*M-chop-F*	CGGGTGGCAGCGACAGAG
*M-chop-R*	CAGGTGTGGTGGTGTATGAAGATG
*M-scd1-F*	AACATTCAATCCCGGGAGAATA
*M-scd1-R*	GAAACTTTCTTCCGGTCGTAAG
*M-fas-F*	TAAAGCATGACCTCGTGATGAA
*M-fas-R*	GAAGTTCAGTGAGGCGTAGTAG
*M-dgat1-F*	CCGATTCTTCCAAGGGAACTAT
*M-dgat1-R*	ATCGTAGTTGAGCACGTAGTAG
*M-cpt1α-F*	ACCGCCACCTCTTCTGCCT
*M-cpt1α-R*	AGTTCCACCTGCTGCTGAG
*M-acc1-F*	CCCAGAGATGTTTCGGCAGTCAC
*M-acc1-R*	GTCAGGATGTCGGAAGGCAAAGG
*M-tfam-F*	GTGAGCAAGTATAAAGAGCAGC
*M-tfam -R*	CTGAACGAGGTCTTTTTGGTTT
*M-pgc1α-F*	GGATATACTTTACGCAGGTCGA
*M-pgc1α-R*	CGTCTGAGTTGGTATCTAGGTC
*M-tnfα-F*	GCCACCACGCTCTTCTGTCTAC
*M-tnfα-R*	GGTTTGTGAGTGTGAGGGTCTGG
*M-il6-F*	ACAAGTCGGAGGCTTAATTACACAT
*M-il6-R*	TTGCCATTGCACAACTCTTTTC

**Table 2:** Different high-fat diets.

		60%HFD		HFD+ FO		HFD + OO	
		Mass Ratio (%)	Energy Ratio (%)	Mass Ratio (%)	Energy Ratio (%)	Mass Ratio (%)	Energy Ratio (%)
protein		23.25	18.14	23.25	18.14	23.25	18.14
fat	Lard oil	34.55	60.64	23.15	40.64	23.15	40.64
	Fish oil	0		11.4	20	0	20
	Olive oil	0		0	0	11.4	0
carbs		27.2	21.22	27.2	21.22	27.2	21.22

## Data Availability

The data that support the findings of this study are available from the corresponding author on reasonable request.

## Supplementary Materials

The following supporting information can be downloaded at: https://www.mdpi.com/article/10.3390/nu15143200/s1, Figure S1: UFAs improve cell viability damage; Figure S2: Effects of the combined use of UFAs and PA on lipid metabolism genes; Figure S3: HFD feeding for 16 w induces obesity and insulin resistance in mice; Figure S4: Multivariate statistical analysis; Figure S5: Expression of antioxidant enzymes in the liver tissue of mice fed UFA diet for 12 w; Figure S6: Results of mice fed with UFA diets (replacing 5% energy of SFAs).

## Abbreviations

T2D	Type 2 Diabetes
NASH	Non-alcoholic steatohepatitis
NAFLD	Non-alcoholic fatty liver disease
EPA	Eicosapentaenoic acid
DHA	Docosahexaenoic acid
ALA	α-linoleic acid
AA	Arachidonic acid
PA	Palmitic acid
SA	Stearic acid
OA	Oleic acid
POA	Palmitoleic acid
LA	Linoleic acid
MUFAs	Monounsaturated fatty acids
PUFAs	Polyunsaturated fatty acids
SFAs	Saturated fatty acids
CCK8	Cell-Counting Kit-8
QC	Quality control
HFD	High-fat diet
IPGTT	Intraperitoneal glucose tolerance test
IPITT	Intraperitoneal insulin tolerance test
ALT	Alanine aminotransferase
AST	Aspartate aminotransferase
LDH	Lactate dehydrogenase
LDL-C	Low-density Lipoprotein cholesterol
HDL-C	High-density lipoprotein cholesterol
FBI	Fasting blood insulin
FBG	Fasting blood glucose
IL-6	Interleukin-6
TNFα	Tumor Necrosis Factor α
ROS	Reactive oxygen species
MDA	Malondialdehyde
DAG	Diglycerides
TC	Total cholesterol
TG	Triglyceride
FO	Fish oil
OO	Olive Oil
PCA	Principal component analysis
OPLS-DA	Orthogonal Partial Least Squares Discriminant Analysis
