# Supplementary material for: Comparison of the Effects of Monounsaturated Fatty Acids and Polyunsaturated Fatty Acids on Liver Lipid Disorders in Obese Mice
Source: Nutrients. 2023 Jul 19;15(14):3200. doi: 10.3390/nu15143200 (PMC10386220; doi:10.3390/nu15143200)
Supplement: Supplementary file 1 [file nutrients-15-03200-s001.zip › nutrients-2474361-supplementary.pdf]

Supplementary Figure

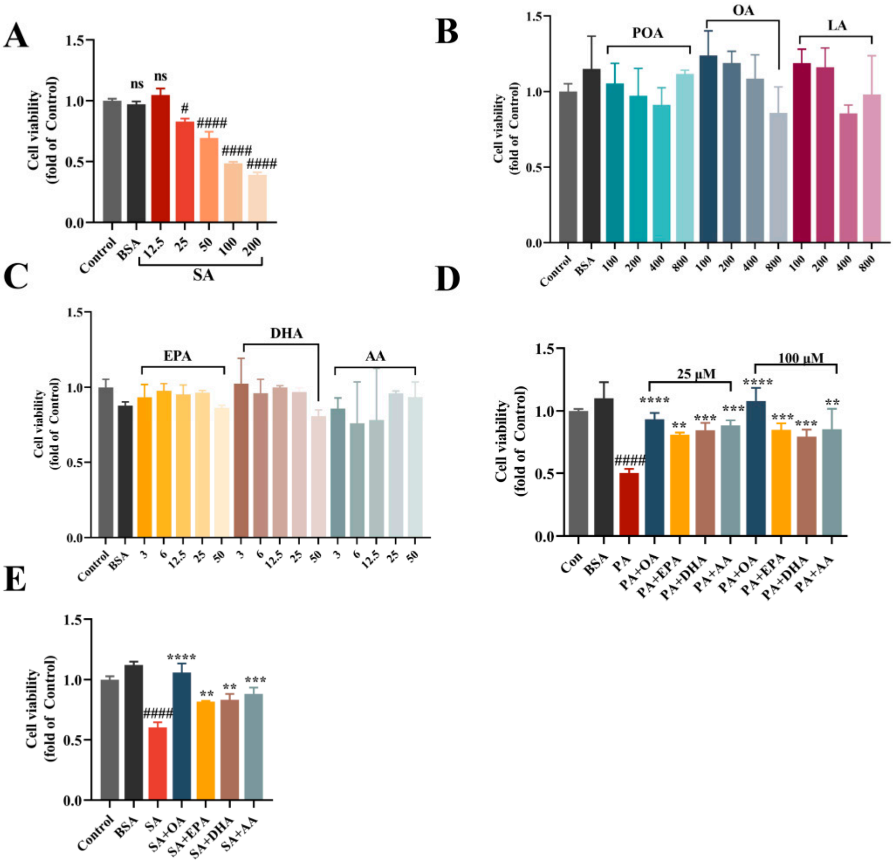

Figure S1. UFAs improve cell viability damage.

(A) Effects of different SA concentrations (12.5 μM~200 μM) incubated for 24 h on the viability of L-02 cells (BSA: 54 μM). (B) Effect of different concentrations of POA (100 μM~800 μM), OA (100 μM~800 μM), LA (100 μM~800 μM), and incubation for 24 h on the viability of L-02 cells (BSA: 216 μM). (C) The effect of different concentrations of EPA (3 μM~50 μM), DHA (3 μM~50 μM), and AA (3 μM~50 μM) on the viability of L-02 cells incubated for 24 h (BSA: 13.5 μM). (D) L-02 cells treated with 25 or 100 μM OA, EPA, DHA, and AA in combination with 100 μM PA (BSA: 27 μM). (E) L-02 cells treated with 100 μM OA and 25 μM EPA, DHA, and AA in combination with 100 μM SA (BSA: 27 μM). ns vs. Control group. $^{\#}P<0.01$, $^{\#\#}P<0.01$, $^{\#\#\#\#}P<0.0001$ vs. BSA group. $^{**}P<0.01$, $^{***}P<0.001$, $^{****}P<0.0001$ vs. PA group. Data are expressed as the mean ± SD, (n≥3). BSA as solvent control.

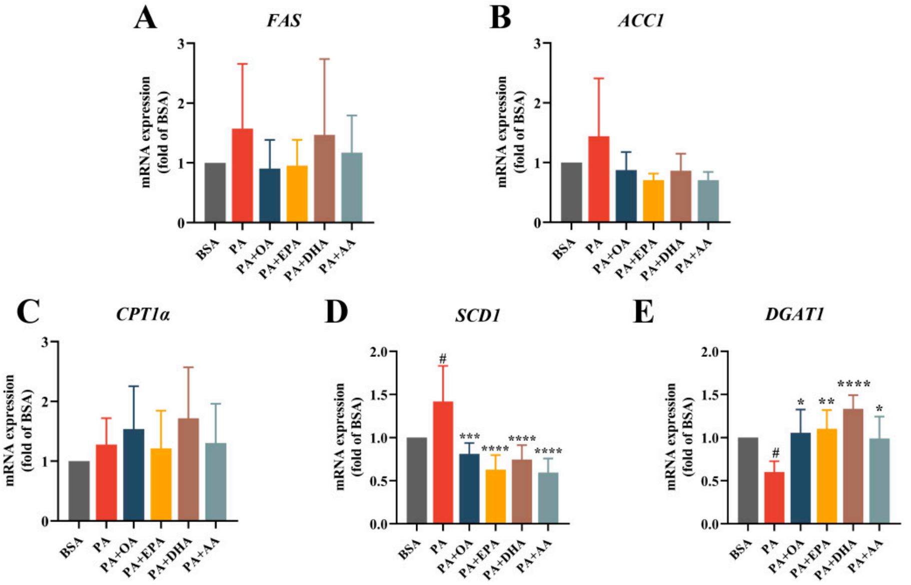

Figure S2. Effects of the combined use of UFAs and PA on lipid metabolism genes. (A) FAS. **(B)** ACC1. **(C)** CPT1α. **(D)** SCD1. **(E)** DGAT1. (BSA: 27 μM; PA: 100 μM; EPA, DHA and AA: 25μM; OA: 100 μM). $^{*}P$<0.05, $^{**}P$<0.01, $^{***}P$<0.001, $^{****}P$<0.0001 vs. PA group. $^{\#}P$<0.05 vs. BSA group. Data are expressed as the mean ± SD, (n≥3). BSA as solvent control.

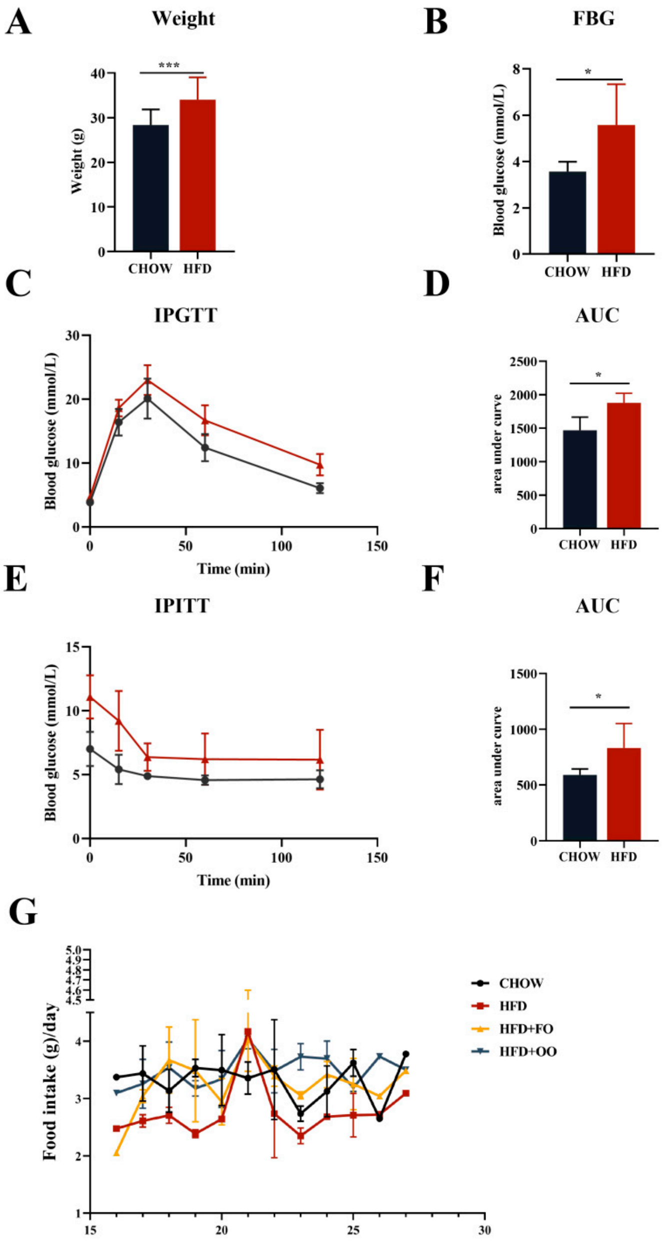

Figure S3. HFD feeding for 16 w induces obesity and insulin resistance in mice.

(A) Weight of mice. (B) FBG. (C) IPGTT. (D) The area under the curve (AUC) of IPGTT. (E) IPITT. (F) AUC of IPITT. (G) Time-related changes in food intake. $^{*}P<0.05$, $^{**}P<0.01$, $^{***}P<0.001$, Data are expressed as the mean ± SD, (n≥5).

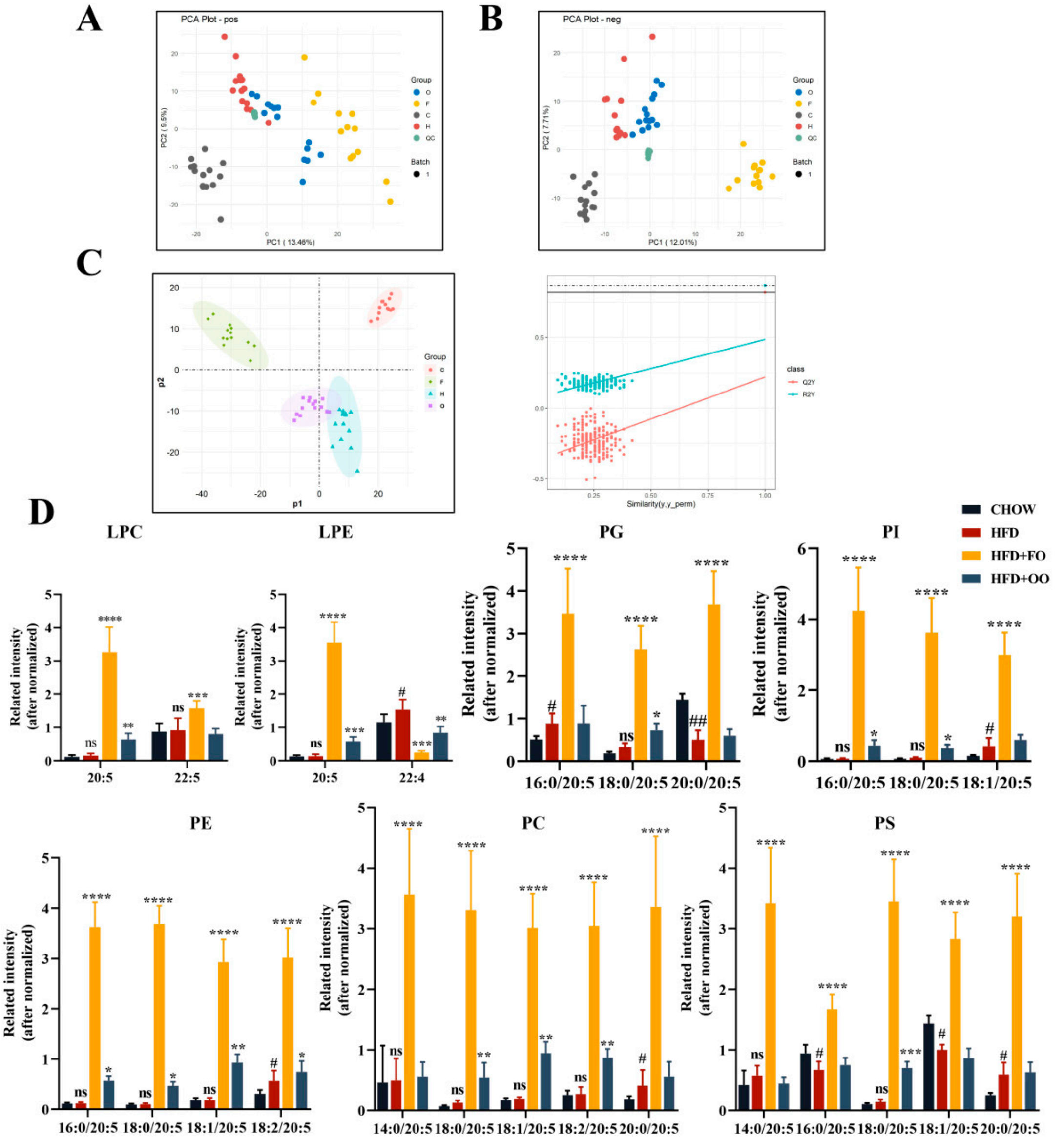

Figure S4. Multivariate statistical analysis.

(A) PCA diagram in positive ion mode. (B) PCA diagram in negative ion mode. (C) PLS-DA (R2X=0.643, R2Y=0.869, Q2=0.819), PLS-DA model for ranking test plots. (D) Significant lipids of FA 20:5. $^{*}P$<0.05, $^{**}P$<0.01, $^{***}P$<0.001, $^{****}P$<0.0001 vs. HFD group. $^{\#}P$<0.05, $^{\#\#}P$<0.01 vs. CHOW group. Data are expressed as the mean ± SD, (n≥13).

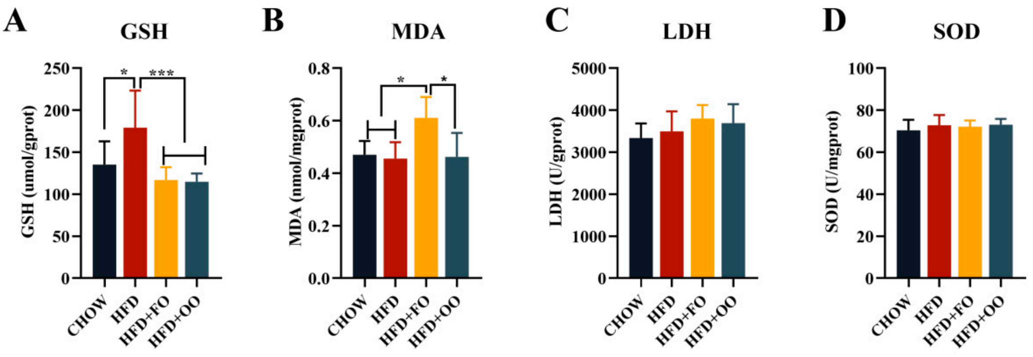

Figure S5.Expression of antioxidant enzymes in the liver tissue of mice fed UFA diet for 12 w. (A) GSH content in liver tissue. (B) MDA content in liver tissue. (C) LDH content in liver tissue. (D) SOD content in liver tissue. $^{*}P<0.05$, $^{***}P<0.001$, Data are expressed as the mean ± SD, (n≥5).

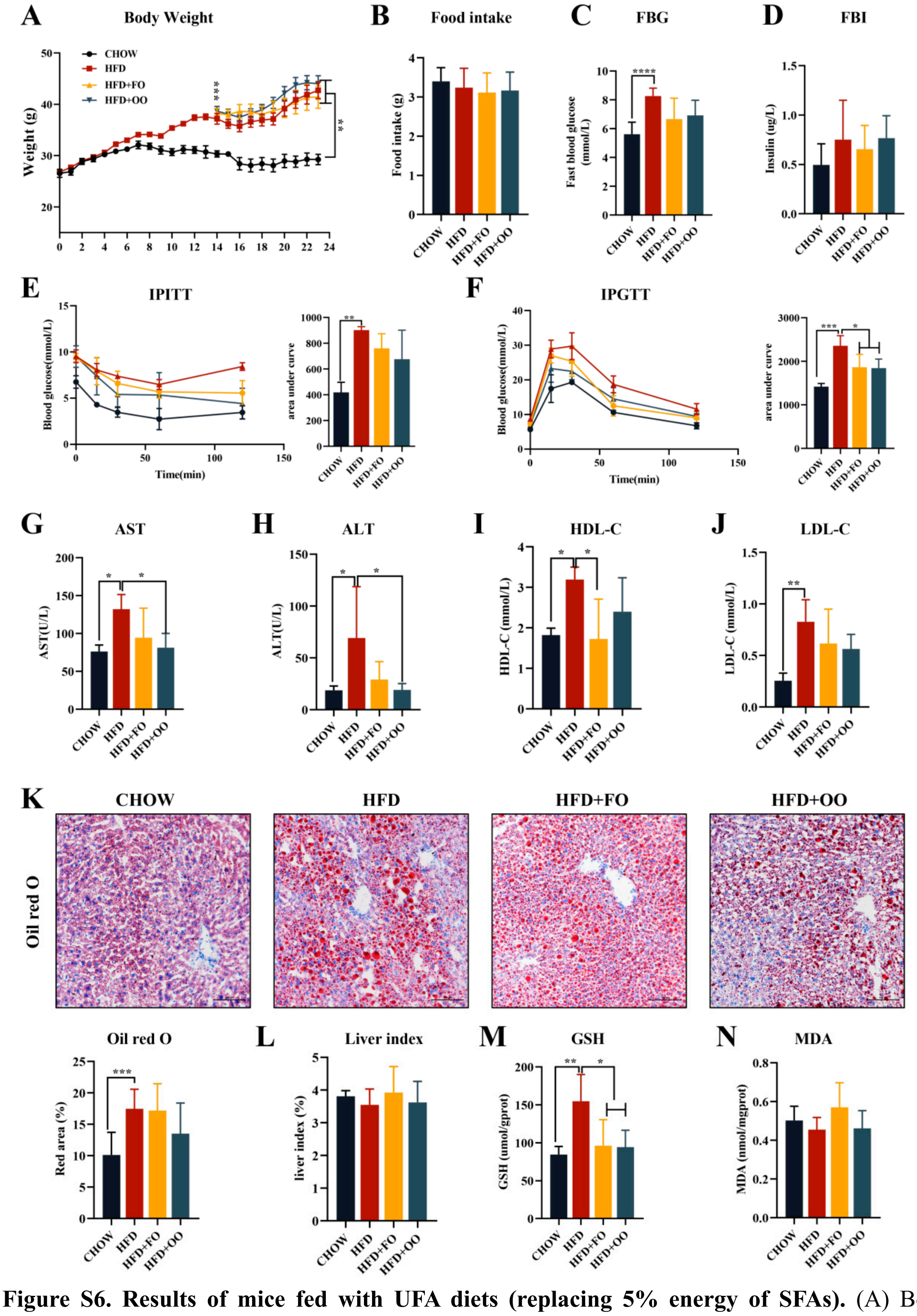

Figure S6. Results of mice fed with UFA diets (replacing 5% energy of SFAs). (A) Body weight. (B) Differences in food intake (g/mouse/day) between groups. (C) FBG. (D) FBI. (E) IPITT. (F) IPGTT. (G) AST. (H) ALT. (I) HDL-C. (J) LDL-C. (K) Oil Red O staining of liver (200X). (L) Liver index.(M) GSH content in liver tissue. (N) MDA content in liver tissue. $^{*}P<0.05$, $^{**}P<0.01$, $^{***}P<0.001$. Data are expressed as the mean ± SD, (n≥5).
